# Intraindividual Lp(a)-Variability in a Real-World Setting

**DOI:** 10.1016/j.jacadv.2026.102601

**Published:** 2026-02-13

**Authors:** Maximilian Seidel, Kamil Rosiewicz, Felix S. Seibert, Moritz Anft, Ulrik Stervbo, Sebastian Bertram, Benjamin Sasko, Christian Ukena, Nina Babel, Timm H. Westhoff

**Affiliations:** a Medizinische Klinik I, Universitätsklinik Marien Hospital Herne, Ruhr-Universität Bochum, Herne, Germany; b Medizinische Klinik II, Universitätsklinik Marien Hospital Herne, Ruhr-Universität Bochum, Herne, Germany

**Keywords:** atherosclerosis, cardiovascular risk, Lp(a), variability

**What is the clinical question being addressed?**

How variable are intraindividual lipoprotein(a) concentrations in real-world clinical practice and is a once-in-a-lifetime measurement sufficient for cardiovascular risk assessment?

**What is the main finding?**

Lp(a) shows substantially higher real-world intraindividual variability than previously assumed, causing frequent risk reclassification and challenging reliance on a single lifetime measurement.

Lipoprotein(a) (Lp(a)) is an independent proatherogenic and prothrombotic cardiovascular risk factor, with its levels correlating with the severity of atherosclerotic cardiovascular disease.^1^ The current guidelines recommend at least a once-in-a-lifetime measurement, based on its presumed high genetic determination. In the past, several trials indeed suggested a variability <10%.^2^ However, a recent subgroup analysis of the OCEAN(a)-DOSE (Olpasiran Trials of Cardiovascular Events And Lipoprotein(a) Reduction — Dose-Finding Study) trial reported a considerably higher intraindividual variability in Lp(a) concentrations.^3^ Whereas the issue of Lp(a) variability was of rather academic interest in the past, it will be of high practical interest in the context of to the upcoming Lp(a) lowering pharmacological therapies. If—in a real-word setting—the intraindividual variability was substantially higher than expected as well, the once-in-a-lifetime recommendation would have to be re-evaluated. To date, however, real-world data on this issue remain heterogeneous, particularly regarding clinical implications. The present study aims to evaluate Lp(a) variability in a real-world setting, analyzing a rather large cohort of patients with a broad spectrum of Lp(a) levels.

## Methods

We performed a longitudinal study including ambulatory patients from a German university hospital. Of 1,163 screened individuals, 992 adults with ≥2 Lp(a) measurements within 48 months were eligible. Patients starting, discontinuing, or switching proprotein convertase subtilisin/kexin type 9 inhibitors during the follow-up, as well as those receiving niacin or undergoing lipoprotein apheresis, were excluded. All measurements were performed in a single accredited laboratory using the Tina-quant Gen.2 assay (analytical repeatability 0.4% to 2.1%). Ethical approval was granted by the Ruhr-University Bochum (15-5279). Mean Lp(a) per patient was calculated. Intraindividual variability (iCV) was defined as SD/mean ×100. Sequential measurement differences were analyzed using Bland-Altman analysis. Lp(a) categories followed consensus thresholds: <75 nmol/L (normal), 75 to 125 nmol/L (borderline), and ≥125 nmol/L (elevated). High variability was defined as >25 nmol/L or >25%. Nonparametric tests were used for comparisons between groups due to non-normal distributions and Spearman correlation was applied to assess associations between Lp(a) concentrations and iCV. *P* < 0.05 was considered statistically significant. Analyses were performed using SPSS Statistics 29 (IBM) and GraphPad Prism 10.

## Results

We analyzed 992 patients with a median of three Lp(a) measurements each (IQR_1-3_: 1-3), totaling 2,849 measurements and 1,857 pairwise differences. The median age was 59 years (IQR_1-3_: 49-68). A total of 593 patients (59.8%) had reported hypertension, 199 (20.1%) diabetes, and 375 (37.8%) had coronary heart disease. The median follow-up was 13 months (IQR_1-3_: 5-27), with a median 6-month interval between measurements (IQR_1-3_: 3-12). The median baseline Lp(a) was 46.0 nmol/L (IQR_1-3_: 11.1-193.8) and across all measurements 48.6 nmol/L (IQR_1-3_: 11.4-194.0). At baseline, 172 patients (17.3%) were below the assay’s limit of detection (<7 nmol/L). During the follow-up, 24 of these (2.4%) developed measurable concentrations (>7 nmol/L), whereas 148 (14.9%) remained below the limit of detection. Overall, 562 patients (56.7%) had normal Lp(a), 66 (6.7%) borderline, and 364 (36.7%) elevated concentrations.

Absolute variation was the highest in those with elevated baseline Lp(a) (median 31.0 nmol/L; IQR: 14.0-65.8), followed by borderline (20.2 nmol/L; IQR: 9.3-39.0) and normal levels (4.7 nmol/L; IQR: 1.2-12.5; *P* < 0.001). In contrast, relative variation was the greatest in those with borderline (19.8%; IQR: 8.8%-39.2%) and normal concentrations (19.7%; IQR: 6.5%-42.0%), and the lowest in patients with elevated values (14.2%; IQR: 6.4%-25.0%; *P* < 0.001). Overall, 112 (11.3%) patients changed risk category between measurements, of whom 69 moved to a higher-risk category: 32 (3.2%) from normal to borderline, 30 (3.0%) from borderline to high, and 7 (0.7%) from low to high.

**Figure 1:**
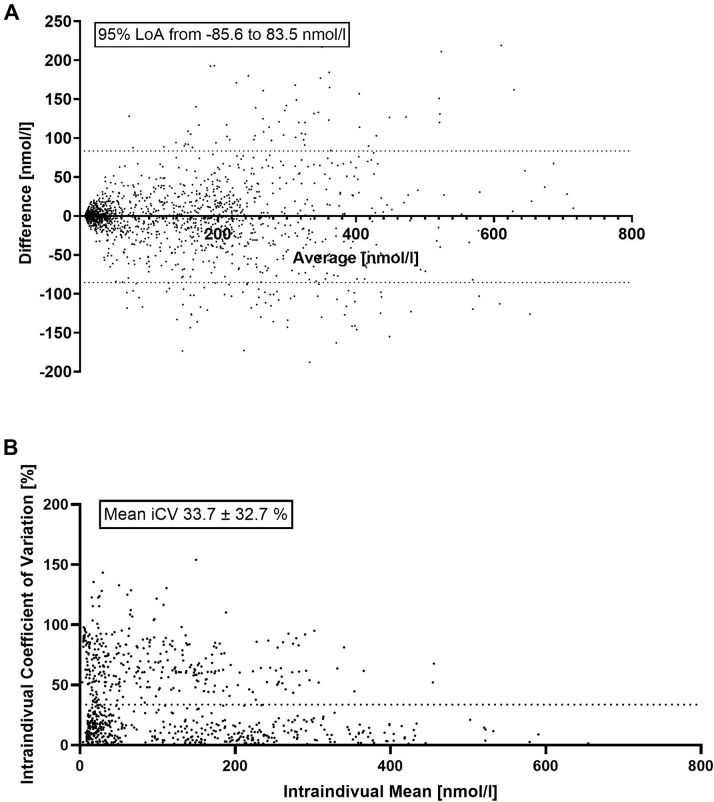
Intraindividual Variability of Lipoprotein(a) Measurements (A) Bland-Altman plot of Lp(a) at baseline vs first follow-up, with 95% limits of agreement (LoA). (B) Intraindividual coefficient of variation (iCV), calculated as the SD of all individual Lp(a) values divided by their mean and multiplied by 100%, shown with mean ± SD.

The median absolute change between measurements was 8.7 nmol/L (IQR: 1.6-30.0), and the median relative change was 14.4% (IQR: 3.7%-31.8%), as displayed in Figure 1A. Bland-Altman limits of agreement spanned −85.6 to +83.5 nmol/L, representing a total fluctuation range of 169.1 nmol/L (Figure 1A). Individual median iCV was 16.3% (IQR: 5.6%-31.3%). Of 1,857 pairs, 512 (27.6%) varied <5%, 249 (13.4%) varied 6 to 10%, 490 (26.4%) varied 11 to 24%, 401 (21.6%) varied 25 to 50%, 114 (6.1%) varied 51 to 75%, 30 (1.6%) varied 76 to 100%, and 61 (3.3%) exceeded 100%.

Among patients without changes in lipid-lowering therapy (n = 389), the median changes remained substantial (6.8 nmol/L and 12.4%). Patients with therapy changes (n = 603) showed slightly higher variability (9.7 nmol/L and 15.2%; *P* = 0.0137). Among 820 patients with measurable values, mean iCV was 33.7% ± 32.7%, as displayed in Figure 1B. Lp(a) concentration weakly predicted iCV (Spearman r = −0.22; 95% CI: −0.29 to −0.16; *P* < 0.001; linear regression R^2^ = 0.06).

## Discussion

In this study, we found a substantially higher iCV in Lp(a) concentrations in a real-world setting than reported in previous clinical trials. Noteworthy, all the measurements took place in the same clinical laboratory. If phase-III trials on Lp(a) lowering agents are positive, a broad variety of antisense oligonucleotides, small interfering RNAs and small molecules will become clinically available shortly. Since all will be approved for predefined Lp(a) thresholds, these findings will become of practical clinical interest.

The 2025 European Society of Cardiology/European Atherosclerosis Society Update defines a threshold of Lp(a) >105 nmol/L as indicative of an elevated cardiovascular risk. With the exception of postmenopausal women, repeated measurements of Lp(a) are not recommended.

In the OCEAN(a)-DOSE placebo analysis, Gaba et al.^3^ reported a median variability of 6% to 10% and an intraindividual coefficient of variation of 10.0% ± 3.9%. However, these results were derived from a highly selected population with markedly elevated Lp(a) (>150 nmol/L), stringent inclusion criteria, and strict control of sample handling, fasting status, and timing. Thus, the low variability observed in this setting may reflect idealized rather than typical clinical conditions. Harb et al.^4^ evaluated 609 subjects with 2 measurements over an 11-year period and observed that 38.1% had an absolute change ≥10 mg/dL, with more than half of individuals in intermediate ranges being reclassified. However, the use of only 2 measurements prevented the assessment of temporal trends or short-term fluctuations, and lipid-lowering therapy use was not controlled, making it difficult to differentiate biological variability from treatment effects. Similarly, Awad et al.^5^ analyzed 11,669 individuals and demonstrated clinically relevant reclassification, particularly among those with borderline concentrations (30-50 mg/dL). Nevertheless, only 2 time points separated by a median of 4.5 years were available, and medication adjustments or acute clinical conditions were not accounted for. These studies collectively support the clinical relevance of repeat testing but do not quantify the magnitude of true biological variability over serial measurements. The present study fills this gap by evaluating multiple measurements per patient under controlled laboratory conditions while still capturing routine clinical heterogeneity.

The present real-world data exceed the iCV of Lp(a) concentrations in clinical trials. Several factors may explain these findings: minor effects of low-density lipoprotein–lowering therapies such as statins on Lp(a), the use of consecutive, rather than mean-based comparisons and greater variability of nutritional and non-nutritional cofactors due to a real-life setting. This study is limited by potential selection bias, as it includes ambulatory patients from a German university hospital and may not reflect Lp(a) variability in the general population.

In conclusion, real-world Lp(a) measurements show substantially greater variability than previously assumed. With the advent of Lp(a)-lowering therapies, this challenges the notion that a once-in-a-lifetime assessment is adequate. Although some variability may reflect changes in lipid-lowering therapy, this is of limited relevance when treatment decisions depend on fixed thresholds. Given the observed reclassification rates and biological within-person variability, a single Lp(a) measurement appears insufficient when therapeutic eligibility is determined by specific cutoffs.

## Funding support and author disclosures

The authors have reported that they have no relationships relevant to the contents of this paper to disclose.
